# Latexin deficiency in mice up-regulates inflammation and aggravates colitis through HECTD1/Rps3/NF-κB pathway

**DOI:** 10.1038/s41598-020-66789-x

**Published:** 2020-06-17

**Authors:** Yaping Li, Baohua Huang, Hua Yang, Shuang Kan, Yanling Yao, Xin Liu, Shiming Pu, Guozhang He, Taj-Malook Khan, Guangying Qi, Zuping Zhou, Wei Shu, Ming Chen

**Affiliations:** 1 State Key Laboratory for Chemistry and Molecular Engineering of Medicinal Resources, School of Chemistry and Pharmacy, Guangxi Normal University, Guilin, 541004 P.R. China; 2 Central Laboratory, The Affiliated Yantai Yuhuangding Hospital of Qingdao University, Yantai, 264000 P.R. China; 3 School of Life Sciences, Research Center for Biomedical Sciences, Guangxi Normal University, Guilin, 541004 P.R. China; 4 School of Basic Medical Science, Guilin Medical University, Guilin, 541004 P.R. China; 5 College of Biotechnology, Guilin Medical University, Guilin, 541004 P.R. China

**Keywords:** Molecular biology, Inflammatory bowel disease

## Abstract

The function of Latexin (LXN) in inflammation has attracted attention. However, no data are available regarding its role in colitis. We report that LXN is a suppressor of colitis. LXN deficiency leads to the severity of colitis in DSS-induced mice, and LXN is required for the therapeutic effect of retinoic acid on colitis. Using a proteomics approach, we demonstrate that LXN interacts and forms a functional complex with HECTD1 (an E3 ubiquitin ligase) and ribosomal protein subunit3 (Rps3). IκBα is one of the substrates of HECTD1. Ectopic expression of LXN leads to IκBα accumulation in intestinal epithelial cells, however, LXN knockdown enhances the interaction of HECTD1 and Rps3, contributing to the ubiquitination degradation of IκBα, and subsequently enhances inflammatory response. Thus, our findings provided a novel mechanism underlying LXN modulates colitis via HECTD1/Rps3/NF-κB pathway and significant implications for the development of novel strategies for the treatment of colitis by targeting LXN.

## Introduction

Inflammatory bowel disease (IBD) is a chronic and non-specific inflammatory disease including two major clinical subtypes, ulcerative colitis (UC) and Crohn disease, characterized by chronic nonspecific inflammation in gastrointestinal tracts^1,2^. Recent studies have shown that the incidence of IBD is increasing year by year, especially in developed countries^3^. For example, more than 1.5 million people in North America suffer from IBD, and an estimated 2 million people in Europe^4^. Although the etiology and pathogenesis of IBD are not fully understood, it is believed that IBD is accompanied by intestinal epithelial integrity loss and immune system disorders^5^. Because IBD has a long course, high recurrence rate and high risk of carcinogenesis, which seriously affects the quality of life of patients, it is of great significance to elucidate the pathogenesis of IBD and find new therapeutic targets.

IBD is usually accompanied by imbalance of pro-inflammatory and anti-inflammatory signaling pathways in the intestine. Increasing evidences suggest that nuclear factor-κB (NF-ĸB) plays a vital role in the pathogenesis of IBD, and the degree of activated NF-ĸB is significantly correlated with the severity of inflammation of the intestine and the colon^6–9^. Various pro-inflammatory cytokines such as IL-1β, IL-6, and TNF-α regulated by NF-ĸB were found increased in IBD patients^6,10^. Latexin (LXN) is the only mammalian carboxypeptidase inhibitor with 222 amino acids in length, which was first identified in the lateral neocortex of rats ^11,12^. Aagaard *et al*.^13^ reported that the expression of LXN in mouse macrophages and mast cells could be induced by lipopolysaccharide (LPS) and colony-stimulating factor 1 (CSF-1), suggesting LXN is implicated in the inflammatory response. This inference was confirmed by You *et al*.^14^, who found that LXN expression inhibited the activity of NF-ĸB by binding to a ribosomal protein subunit 3 (Rps3). Interesting is that Rps3 has been reported to interact with many proteins including IKK^15^, NF-κB^16^ and IκBα^17^, indicating the critical role of LXN in NF-κB signaling pathway, however, the mechanism remains largely elusive, and no data are available regarding the role of LXN in colitis.

In this study, we investigated the contribution of LXN on the development of colitis. We found that LXN deficiency accelerates the process of dextran sulfate sodium (DSS)-induced colitis in mice. The mechanisms underlying LXN forms a complex with HECTD1, IκBα and Rps3, and this complex further regulates IĸBα ubiquitylation and thus regulates the inflammatory response mediated by NF-κB. Our study provides a novel mechanism by which LXN modulates colitis, suggesting LXN is a potential target for IBD treatment.

## Materials and Methods

### Cell and reagents

HEK293T, HIEC and HCT116 cells were purchased from ATCC, and cultured in Dulbecco’s modified Eagle’s medium (DMEM) in a humidified atmosphere of 5% CO_2_ at 37 °C. Antibodies used in this study were shown in Supplementary Table S1. HA-HECTD1 plasmid was purchase from BioVector NTCC Inc (Beijing, China); Ub-K63R and Ubiquitin-K48R plasmids were purchased from Addgene; His-IκBα was purchase from Beyotime Biotechnology (Nantong, China). Dextran sulfate sodium (DSS: CAS 9011-18-1, MW: 36 to 50 kDa) was purchased from Yeasen Biotech Co., Ltd (HongKong, China).

### Mouse model and ethics statement

Homozygous LXN^−/−^ mice  (KO) were maintained as F10 generations from heterozygous LXN^+/−^ mice (B6N.129S2-Lxn<tm1Yari > /YariRbrc), which were generated and purchased from RIKEN BioResource Research Center (Japan)^18^, and fed under specific pathogen-free conditions. Acute dextran sulfate sodium (DSS) colitis was induced in wild type (WT) and LXN^−/−^ mice according to the previously published method with minor modification^19^. Briefly, WT and LXN^−/−^ male mice (10 weeks old) received 2.5% (w/v) DSS in their drinking water for 7 days and followed by 8 days of DSS-free water to recovery. Fresh DSS solution was provided every day. Control mice drank only distilled water. Retinoic acid (RA) treatment experiment was divided into four groups: (1) WT + DSS, (2) KO + DSS, (3) WT + DSS + RA, (4) KO + DSS + RA. The mice of DSS groups were fed 2.5% (w/v) DSS dissolved in the drinking water on day 0, RA (20 mg/kg) was administered intraperitoneally (i.p.) since day 3 and repeated daily until the mice were killed on day 8. Disease symptoms of colitis were assessed daily by measurement of body weight, evaluation of stool consistency and detection of bloody stools. Animals were sacrificed by inhalation of CO_2_. Colons were excised and measured for length. All studies involving animals were performed according to the guidelines for the Care and Use of Laboratory Animals (Laboratory Animal Center of Guangxi Normal University). All animal experiment protocols were approved by the Ethics Review committee for Lab Animal Use at Guangxi Normal University.

### MS/MS analysis

Cells were lysed by NP-40. Then the cell lysates were immunoprecipitated with anti-LXN antibody. The bound proteins were analyzed by sodium dodecyl sulfate-polyacrylamide gel electrophoresis (SDS–PAGE) and Coomassie brilliant blue staining. Gel slices were excised and subjected to in-gel digestion with trypsin. Tryptic peptides were spotted using the Tempo™ LC-MALDI Spotting System (AB SCIEX, USA) and analyzed using MALDI-TOF/TOF 5800 mass spectrometer (AB SCIEX, USA). All MS/MS spectra were searched against the database (2017_03 released UniProtKB/Swiss-Prot human database, 20,183 entries). Proteins with high-confidence (FDR < 0.01) were considered as positively identified proteins.

### Western Blot

Cells were lysed using RIPA buffer (25 mM Tris-HCl, 150 mM NaCl, 1% Nonidet P-40, 1% sodium deoxycholate, 0.1% SDS, pH 7.6, and proteinase inhibitor mixture). Protein samples were resolved by SDS-PAGE and transferred to nitrocellulose (BioRad). Blots were developed with diluted antibodies.

### H&E and Immunofluorescence staining

The colon was fixed using 4% paraformaldehyde and embedded in paraffin. For H&E staining, 5 µm sections were sliced from the paraffin block and stained with hematoxylin and eosin. For immunofluorescence staining, cells were fixed in 4% formaldehyde in PBS. Fixed cells were incubated with primary antibody as indicated, followed by fluorescein-5-isothiocyanate (FITC)-conjugated secondary antibodies (1:250 dilution, Invitrogen) and Tetramethylrhodamine isothiocyanate (TRITC)-conjugated secondary antibodies (1:300 dilution; Invitrogen). Cell nuclei were stained with DAPI. Images were visualized using an EVOS microscopy (EVOS™ FL Auto 2, ThermoFisher).

### Enzyme linked immunosorbent assay (ELISA)

Snap-frozen colon tissue was homogenized in ProcartaPlex cell lysis buffer (Affymetrix, San Diego, CA, USA), and total protein was quantified using the Pierce BCA Protein Assay Kit (Fisher Scientific). Before mice was sacrificed, blood was taken from the orbital venous plexus after ether deep anesthesia. Cytokine levels in serum and colon tissue were quantified by using ELISA kit according to the manufacturer’s protocol. ELISA assay for mice TNF-α (Catalog number, E-EL-M0049c), IL-1β (Catalog number, E-EL-M0037c), IL-6 (Catalog number, E-EL-M0044c), and IL-12 (Catalog number, E-EL-M0726c) in serum and colon lysate were undertaken following the manufacturer’s instructions (Elabscience, Wuhan, China). The measurement was performed in triplicate.

### Luciferase Reporter Assay

HEK293T cells were transfected with 200 ng pNF-κB-Luc and 50 ng of Renilla luciferase reporter plasmid pRL-SV40 (Beyotime Biotechnology, Nantong, China) using PEI transfection reagent. 48 h after transfection cells were lysed, and the reporter activity was determined with a luminescence counter using the Dual-Luciferase Reporter Assay System (Beyotime Biotechnology, Nantong, China) according to the manufacturer’s instructions.

### Real-time quantitative PCR

Total RNAs were extracted from cells or tissues by using TRIzol Reagent (Life Technologies, Rockville, MD) according to the manufacturer’s instructions. qRT-PCR was performed on cDNA from 200 ng of total RNA by using cDNA Synthesis kit and SYBR® Green Master Mix Kit (Exqion). The primer sequences are described in Supplementary Table S2.

### RNA interference

Control siRNA and the on-target individual siRNAs were purchased from Sigma (Supplementary Table S3). siRNA was transfected into cells with Lipofectamine RNAiMAX transfecting reagent (Invitrogen) in a serum-free medium according to the manufacturer’s recommendation. The knockdown efficiency was verified by quantitative PCR or Western blot.

### Statistical analyses

All experiments were performed at least 3 times. Data are expressed as means ± SEM. All of the data were evaluated using Prism 8.0 (GraphPad software). Statistical significance between two groups was evaluated using the Student’s t test (unpaired, two-tailed), a value of *p* < 0.05 was considered statistically significant. The survival curve is drawn by GraphPad and analyzed by using the long-rank test.

## Results

### LXN deficiency leads to the severity of colitis induced by DSS

**Figure 1:**
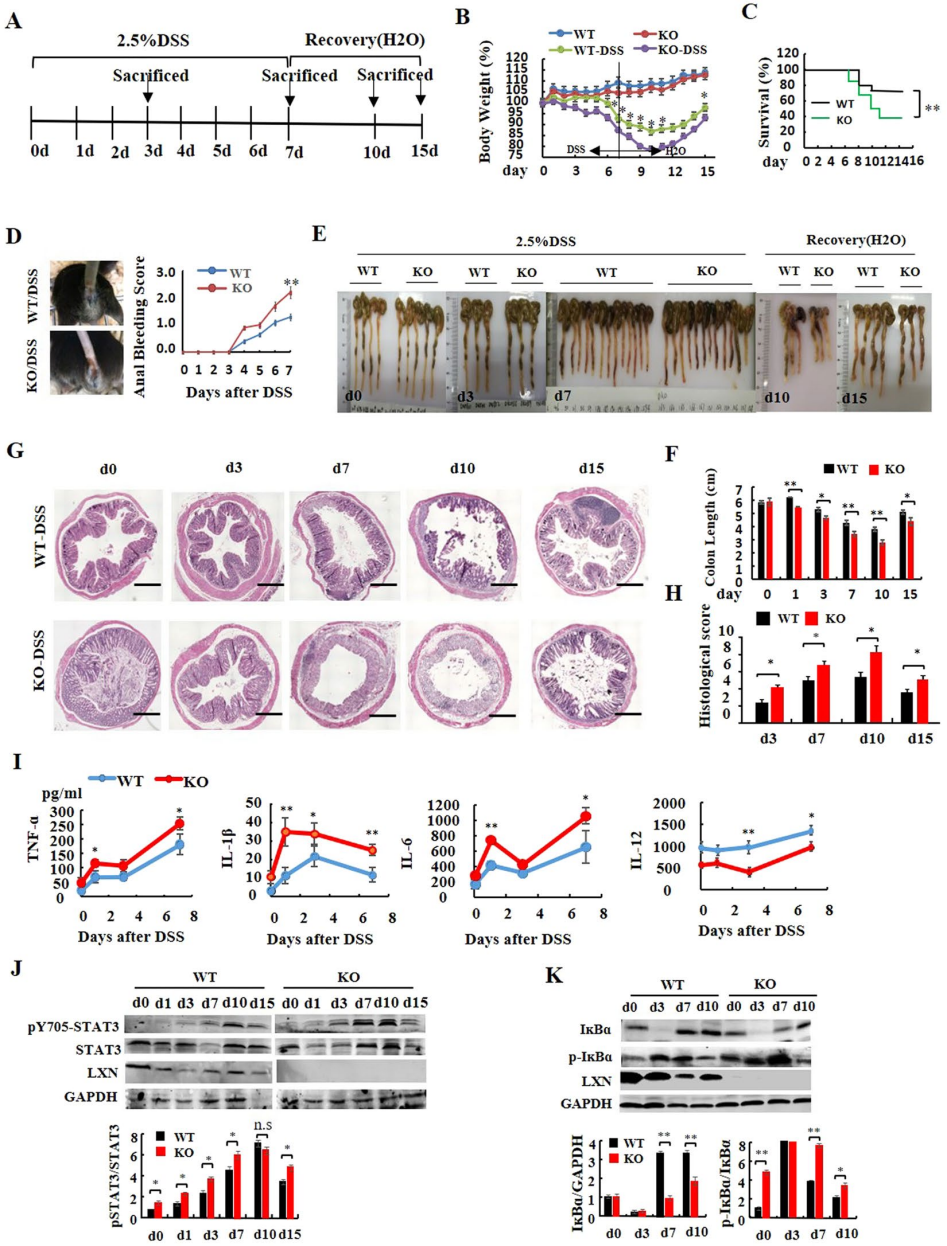
LXN deficiency accelerates DSS-induced colitis in mice. (**A**) Experimental protocol for the induction of the acute colitis model in LXN^−/−^ (KO) and littermate LXN^+/+^ (WT) mice. **(B)** Changes in body weight of WT and KO mice (relative to starting weight, set as 100%) is shown (n = 16). (**C**) Kaplan–Meier survival plot of WT and KO mice after induction with 2.5% DSS. (**D**) Anal bleeding is photograph and bleeding score is shown (n = 16). (**E**) The gross morphology of shortened colon was photograph. (**F**) The colon length was measured at the time point as indicated (n = 6). (**G**,**H**) Representative images of H&E-stained colons from WT and KO mice treated or untreated with DSS for different days are shown. Scale bars = 100 μm. The histological score of WT and LXN KO mice during DSS-mediated colitis at day 3, day 7, day10 and day15 are shown (**G**). (**I**) Data represent levels of cytokines indicated in serum from WT and KO mice treated with DSS for different days (n = 6). (**J**,**K**) Protein levels of LXN, STAT3, p-STAT3 (J), IĸBα and p-IĸBα (K) are detected in colon tissue from WT and KO mice after induction with 2.5% DSS. The results shown are the mean ± SEM. The survival curve is analyzed by using the long-rank test, other data was evaluated using the two-tailed unpaired t-test. Data are representative of three independent experiments. *p < 0.05; **p < 0.01; n.s, no significant.

To examine the effect of LXN on colitis, LXN^−/−^ mice was generated (Fig. S1). We used 2.5% DSS for 7 d to induce colitis, and followed by 8 days of DSS-free water to recovery (Fig. 1A). At indicated time points, mice were euthanized, and the colon was excised and measured. LXN^−/−^ mice had normal increases in body weight and colon length compared with WT mice. We found that 2.5% DSS treatment LXN^−/−^ mice developed more severe clinical symptoms of inflammatory bowel disease (IBD), including rapid weight loss (Fig. 1B), low survival rates (Fig. 1C), severe bloody stools (Fig. 1D), severe splenomegaly (Fig S2) and diarrhea (data not shown). Histological analysis of the colons revealed more severe disease, shortened colon (Fig. 1E,F), and a disruption of mucosal structures (Fig. 1G,H) in LXN^−/−^ mice. As shown in Fig. 1G, for WT mice, histological examination showed the colon structure was normal after 3 days of DSS treatment, and partially loss of crypt structure, epithelial cell and tissue destruction at day 7 of DSS treatment; for LXN^−/−^ mice, we found that the crypt structure destroyed began to appear at day 3 of DSS challenge, and the crypt structure and epithelial cells were completely lost at day 7 and lasted until the day 10 (Fig. 1G).

We further examined the production of inflammatory cytokines in serum (Fig. 1I) and colon tissues (Fig. S3) from WT and LXN^−/−^ mice during DSS-induced colitis. We found that the levels of TNF-α, IL-1β, and IL-6 were significantly increased, however, the production of IL-12 was remarkably decreased in LXN^−/−^ mice during DSS-induced colitis, suggesting TNF-α, IL-1β, IL-6 and IL-12 involved in DSS-induced colitis in LXN^−/−^ mice. Western blotting showed the protein level of LXN was decreased; however, the phosphorylation of signal transducer and activator of transcription3 (STAT3) was increased during colitis (Fig. 1J). Interestingly, we fund that the phosphorylation of STAT3 in LXN^−/−^ mice was significantly increased compared with that in WT mice (Fig. 1J). It should be noted that the expression of IκBα was generally decreased in LXN^−/−^ mice, while the phosphorylation of IκBα was significantly increased, especially on the day 7 of DSS treatment (Fig. 1K).

### LXN attenuates TNF-α-induced inflammatory response

**Figure 2:**
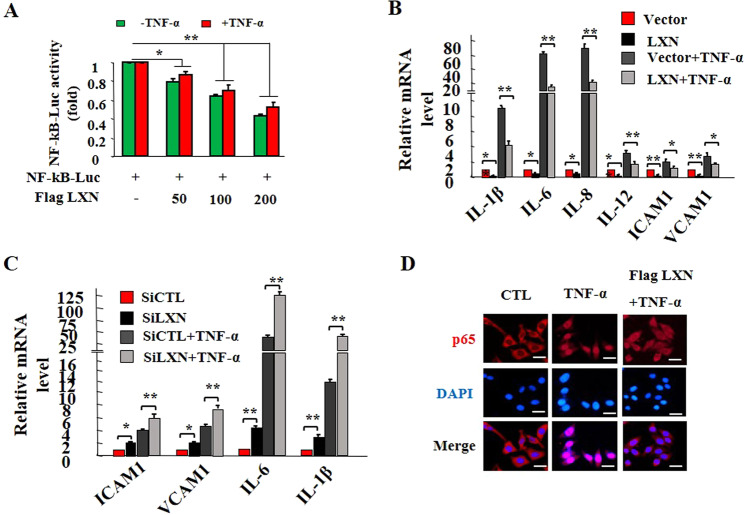
LXN attenuates inflammatory response in HIECs. (**A**) HEK293T cells cultured in 6-well plates were co-transfected with 200 ng NF-κB-Luc reporter plasmid and 0, 50 ng, 100 ng and 200 ng of Flag-LXN as indicated, and 50 ng RL-SV40 was used as a control. 48 h after transfection, cells were treated with TNF-α (20 ng/mL) for 6 h, and then cells were lysed, and the reporter activity was determined with a luminescence counter using the Dual-Luciferase Reporter Assay System. (**B**) HIEC cells were seeded in 6-well plates 48 h after transfection of Flag-LXN or control plasmid, the cells were stimulated with TNF-α (20 ng/mL) for 6 h, and the expression of IL-1β, IL-6, IL-8, IL-12, ICAM1 and VCAM1 transcripts was examined by qPCR. (**C**) HIEC cells were treated with LXN siRNA for 72 h, the cells were stimulated with TNF-α (20 ng/mL) for 6 h, and the expression of IL-1β, IL-6, ICAM1 and VCAM1 transcripts was examined by qPCR. (**D**) HIEC cells were seeded in 6-well plates 48 h after transfection of Flag-LXN or control plasmid, the cells were stimulated with TNF-α (20 ng/mL) for 30 min, the cells were then washed, fixed, and stained with anti-p65 antibody. Scale bars = 10 μm. Data are representative of three independent experiments. *p < 0.05; **p < 0.01.

LXN has been reported involving an inflammatory process in many cell lines and diseases^13^. We first tested the effects of LXN on NF-κB mediated inflammatory pathway. Luciferase reporter assay showed that over-expression of LXN markedly down-regulated NF-κB-Luc activity both in basal and TNF-α stimulation with a dose-dependent manner (Fig. 2A). This was further supported by qPCR analysis of cytokines in HIECs (Fig. 2B,C). We found that overexpressed LXN significantly attenuates the expression of cytokines (IL-1β, IL-6, IL-8, IL-12), and cell adhesion molecules (ICAM1 and VCAM1), which were dramatically induced by TNF-α stimuli (Fig. 2B). LXN knockdown further increases TNF-α-induced cytokine expression (Fig. 2C). Consistent with these observations, we found that TNF-α stimulation promotes NF-κB p65 subunit nuclear translocation; however, this process was inhibited by overexpression of LXN (Fig. 2D). Together, we concluded that LXN attenuates TNF-α-induced inflammatory response, at least in part, through inhibition of p65 activity.

### LXN regulates IĸBα accumulation in cells

**Figure 3:**
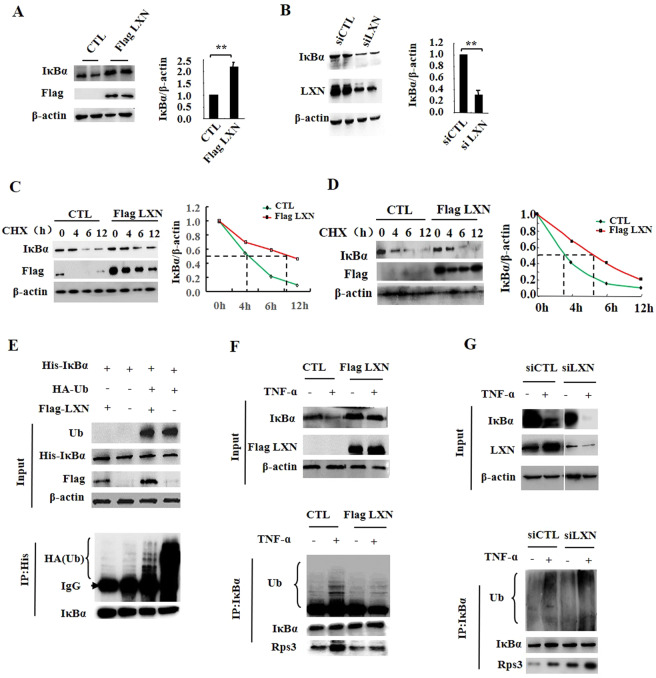
Effect of LXN on ubiquitous degradation of IκBα. (**A**) HIEC cells were seeded in 6-well plates, 48 h after transfection of Flag-LXN or control plasmid, the cells were lysed and the expression of IĸBα and Flag-LXN were determined by Western blot and quantitated by image J. (**B**) HIEC cells were seeded in 6-well plates, 72 h after transfection of siLXN or siCTL, the cells were lysed and the expression of IĸBα and LXN were determined by Western blot and quantitated by image J. (**C,D**), HIEC (**C**) and HCT116 (**D**) cells seeded in 6-well plates were transfected with pFlag-LXN plasmid. 48 h after transfection, cells were treated with CHX (100 µg/mL) for 0, 4, 6 and 12 h, cell lysates were harvested, and IκBα was determined by western blot. Quantitative analysis of IκBα was performed by image J. **(E)** HEK293T cells were co-transfected with His-IκBα, Flag-LXN and HA-Ub plasmids as indicated. For ubiquitination assay, the cells were incubated in the presence of 10 μM MG132 for 12 h before assay. 48 h after co-transfection, immunoprecipitation was performed with anti-His antibody, and HA-antibody was used to detect the ubiquitylation of IκBα. (**F**) HCT116 cells were transfected with Flag-LXN plasmid. 48 h after transfection, cells were treated with TNF-α (20 ng/mL) for 30 min, immunoprecipitation was performed with anti-IκBα antibody and then separated by 10% SDS-PAGE, followed by immunoblotting with antibody as indicated. **G**, **H** CT116 cells were transfected with siLXN or siCTL for 72 h, and then the cells were stimulated with TNF-α (20 ng/mL) for 30 min, immunoprecipitation was performed with anti-IκBα antibody, followed by immunoblotting with antibody as indicated. Data are representative of at least three independent experiments. The results shown are the mean ± SEM. A two-tailed unpaired t-test was used to compare experimental groups. *p < 0.05; **p < 0.01.

In most cell types, IκBα controls the immediate-early activation of NF-κB in TNF-α induced inflammatory response via its degradation^20^. We therefore examined whether the stability of IκBα was modulated by LXN. We found that the protein level of IκBα in HIEC cells was significantly increases by ectopic expression of LXN (Fig. 3A), however, was markedly decreased when LXN siRNA transfected (Fig. 3B). Cycloheximide (CHX)-based protein stability assays revealed that the stability of IκBα was dramatically increased when LXN plasmid was transfected (Fig. 3C,D). Taken together, these findings indicate that ectopic expression of LXN leads to IκBα accumulation in intestinal epithelial cells.

### LXN and Rps3 involve in IκBα ubiquitylation, and LXN attenuates TNF-α-induced interaction between Rps3 and IκBα

Since the ubiquitin-proteasome pathway plays a crucial role in the canonical pathway of NF-κB activation, the inhibitory for IκBα degradation by ectopic expression of LXN prompted us to ask whether LXN directly inhibits the ubiquitylation of IκBα. To test this hypothesis, His-tagged IκBα, HA-tagged Ub, and Flag-tagged LXN plasmids were co-transfected into 293 T cells. We found that HA-tagged Ub lead to the significant ubiquitylation of IκBα, however, co-expression of LXN caused a reduction in IκBα ubiquitylation (Fig. 3E). We further shown that TNF-α induced a rapid IκBα ubiquitylation in HCT116 cells, and the ubiquitylation of IκBα were markedly inhibited by ectopic expression of LXN (Fig. 3F), however, dramatically promoted by LXN knockdown (Fig. 3G). We also detected the phosphorylation of IĸBα and IKK. The results show that ectopic expression of LXN can indeed inhibit the TNF-α induced phosphorylation of IĸBα, but has no effect on the phosphorylation of IKK (Fig. S4), which indicates that overexpression of LXN does not affect the activation of IKK, and indicating that the inhibition of phosphorylation of IĸBα may be due to the inhibition of the interaction between IKK and IĸBα by overexpression of LXN. Interestingly, we found that the interaction between IκBα and Rps3 could be induced by TNF-α stimuli, which was further enhanced when LXN was knockdown (Fig. 3G, lower panel), however, dramatically blocked by ectopic expression of LXN (Fig. 3F, lower panel), suggesting the potential role of Rps3 in IκBα ubiquitylation process, at least in part, via interaction with IκBα.

### LXN forms a complex with HECTD1 and Rps3, and LXN deficiency increases the interaction between HECTD1 and Rps3

**Figure 4:**
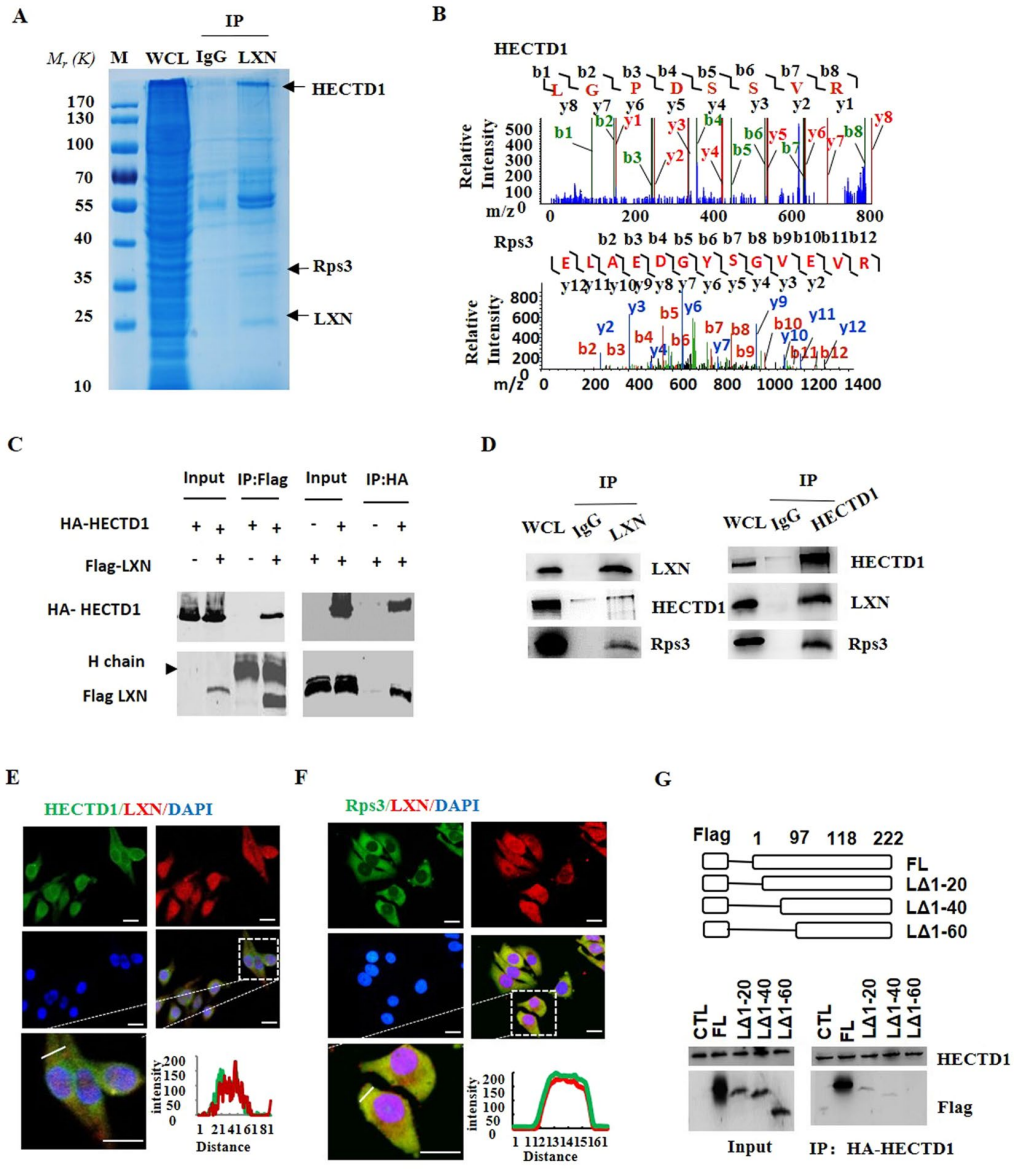
LXN forms a complex with HECTD1 and Rps3 in HIEC cell. (**A,B**) Cell lysates from HIEC cells were immunoprecipitated with anti-LXN antibody or control IgG, and then the purified protein complex was separated by 10% SDS-PAGE and stained with coomassie brilliant blue (**A**), the differential bands were cut and in-gel digested with trypsin. Peptides were subject to LC-MALDI MS/MS assay. Identified peptide and MS/MS spectrum that matched with the sequence of HECTD1 and Rps3 are shown (**B**). **(C)** HEK293T cells were co-transfected with HA-HECTD1 and Flag-LXN plasmids. 48 h after co-transfection, immunoprecipitation was performed with either anti-Flag antibody or anti-HA antibody to detect the interaction of HECTD1 with LXN. (**D**) Cell lysates obtained from HIEC cells were immunoprecipitated with anti-LXN, anti-HECTD1 antibody, respectively, IgG as control antibody, and then separated by 10% SDS-PAGE. Transferred membrane was immunoblotted with antibodies as indicated. **(E**,**F)** HCT116 cells were cultured in 6-well plates. The localization of HECTD1 and LXN (**E**), Rps3 and LXN (**F**) were analyzed by immunofluorescence staining with HECTD1, Rps3 and LXN antibody. Higher magnification inserts show details of localization. The pixel intensity of localization in the selected regions (white line) are shown. Scale bars = 10 μm. (**G**) HA-HECTD1 expression vector in combination with either empty vector or expression vectors of Flag-LXN mutants were co-transfected into HEK293T cells. Extracted proteins were precipitated by anti-HA antibody, and then separated by 10% SDS-PAGE. The transferred membrane was immunoblotted with either anti-Flag or anti-HA antibody.

**Figure 5:**
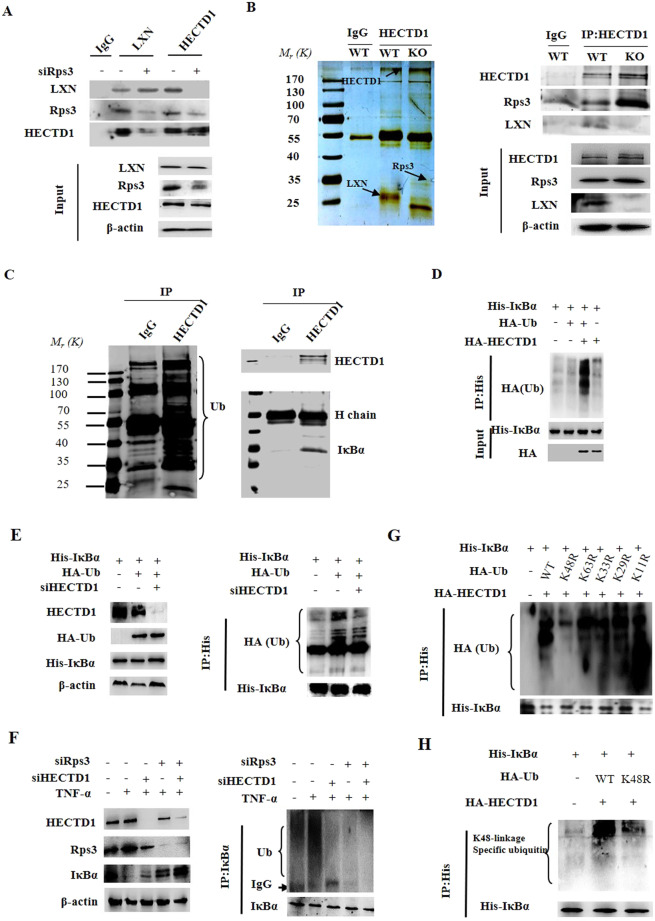
Rps3 is required for HECTD1 to ubiquitinate IκBα. (**A**) HIEC cells were transfected with siRps3 for 72 h, and then the cell lysates were obtained for immunoprecipitation with anti-LXN and anti-HECTD1 antibody, respectively. The immunoblotting was performed with antibody as indicated. (**B**) Protein samples from LXN^+/+^ (WT) and LXN^−/−^ (KO) mice colon tissue were immunoprecipitated with anti-HECTD1 and anti-IgG antibody, and then the complex was separated by 10% SDS-PAGE, and stained with silver staining (left panel). Transferred membrane was immunoblotted with antibodies as indicated (right panel). (**C**) Cell lysates from HCT116 cells were immunoprecipitated with anti-HECTD1 antibody or control IgG, and then the purified protein complex was separated by 10% SDS-PAGE. Transferred membrane was immunoblotted with anti-ubiquitin (left panel), anti-HECTD1 and anti-IkBα antibodies, as indicated (right panel). (**D**) HEK293T cells were co-transfected with His-IκBα, HA-Ub and HA-HECTD1 plasmids as indicated. For ubiquitination assay, the cells were incubated in the presence of 10 μM MG132 for 12 h before assay. 48 h after co-transfection, immunoprecipitation was performed with anti-His antibody, and ubiquitylation was detected by Western blot. (**E**) HCT116 cells were co-transfected with His-IκBα, HA-Ubiquitin plasmid plus HECTD1 siRNA for 72 h. Cell lysates were subjected to immunoprecipitation with anti-His antibody, and then the ubiquitylation of His-IκBα was determined by western blot. (**F**) 72 h after transfection of either HECTD1 siRNA or Rps3 siRNA, HCT116 cells were stimulated with TNF-α (20 ng/mL) for 30 min, cell lysates were subjected to immunoprecipitation with anti-IκBα antibody followed by immunoblot analysis with anti-Ub and anti-IκBα. (**G**) HEK293T cells were co-transfected with His-IκBα, HA-HECTD1, wild-type HA-Ub (WT) or Ub mutant plasmids as indicated. 48 h after transfection, cell lysates were immunoprecipitated with anti-His antibody and subjected to immunoblotting with anti-HA antibody. (**H**) HEK293T cells were co-transfected with His-IκBα, HA-HECTD1, wild-type HA-Ub (WT) or Ub mutant plasmids (K48R). 48 h after transfection, cell lysates were immunoprecipitated with anti-His antibody and subjected to immunoblotting with K48-linkage specific ubiquitin antibody. Data are representative of three independent experiments.

To further explore the mechanism how LXN regulates IκBα ubiquitylation, we have undertaken a proteomic screen to identify intracellular targets of LXN. We indeed identified HECTD1, an E3 ubiquitin ligase, as a potential partner of LXN in HIEC cells (Fig. 4A,B). We first validated the interaction of HECTD1 and LXN in 293 T cells by co-transfected with Flag-LXN and HA-HECTD1(Fig. 4C). In addition, pull-down assays showed that LXN interacted endogenously with HECTD1 and Rps3 in HIEC cells (Fig. 4D). These interactions were further confirmed by immunostaining (Fig. 4E,F). We further want to determine the critical region in LXN for HECTD1 association. To this end, we constructed a series of Flag-tagged LXN deletion mutants, and expressed these proteins in 293T cells. Cell lysates were then immunoprecipitated with anti-HECTD1 antibody, and analyzed by western blot. We found that deletion of the N-terminal region of LXN residues 1-20 (LΔ1-20) and 1-40 (LΔ1-40) significantly attenuate the association of LXN and HECTD1. However, deletion of 1-60 (LΔ1-60) completely abolished its interaction with HECTD1 (Fig. 4G). Thus, these findings indicate that the N-terminal region in LXN is critically important for interaction with HECTD1. Therefore, our data clearly demonstrated that LXN, HECTD1, and Rps3 form a complex in colon cells. Interestingly, we found that Rsp3 knockdown attenuates the interaction between LXN and HECTD1 (Fig. 5A). On the contrary, LXN deficiency enhances the interaction of HECTD1 and Rps3 in LXN^−/−^ mice colon tissue (Fig. 5B), indicating Rps3 is required for the interaction between LXN and HECTD1, as well as, LXN and HECTD1 compete for Rps3.

### HECTD1 catalyzes the Lys48-linked polyubiquitination of IκBα, and Rps3 is required for HECTD1 to target IκBα

HECTD1 is an E3 ubiquitin ligase, we therefore ask whether HECTD1 is involved in IκBα ubiquitylation. Immunoprecipitation was performed in HCT116 cells. We found that IĸBα was detected in HECTD1 complex (Fig. 5C), indicating IĸBα may be a substrate for HECTD1. We further examined the ubiquitylation of IκBα in 293 T cells co-transfected with HA-IκBα, His-Ub, and HA-HECTD1. We found that ectopic expression of HA-HECTD1 markedly induced the ubiquitylation of IκBα (Fig. 5D). The role of endogenous HECTD1 in HCT116 cells was determined by expressing HECTD1 siRNA, and then His-IκBα and HA-Ub plasmids were co-transfected. We observed that polyubiquitinated His-IκBα was detected in the presence of HA-Ub; however, the IκBα ubiquitylation impaired in HECTD1 knockdown cell (Fig. 5E, right panel). Similar results were obtained when we evaluated the role of HECTD1 in TNF-α induced IκBα ubiquitylation (Fig. 5F), indicating the functional role of endogenous HECTD1 in IκBα ubiquitylation. Likewise, the ubiquitylation of IκBα was markedly reduced in Rps3 knockdown cells (Fig. 5F). Considering the association between IκBα and Rps3 were significantly enhanced by TNF-α stimuli (Fig. 3F,G). Therefore, we concluded that Rps3 is required for HECTD1 to target and ubiquitylate IκBα; LXN knockdown promotes the interaction between Rps3 and HECTD1, thus contributing to the ubiquitination of IκBα, and subsequently enhances inflammatory response. Most importantly, the type of polyubiquitin chain catalyzed by HECTD1 was determined. Our data revealed that the replacement of Lys48 with Arg in HA-Ub markedly reduced the ubiquitylation of IκBα (Fig. 5G), indicating HECTD1 catalyzed the Lys48-linked polyubiquitination of IκBα, which was further confirmed by detecting with K48-linkage specific ubiquitin antibody (Fig. 5H).

### Retinoic acid represses TNF-α-induced inflammatory response by pharmacological induction of endogenous LXN

**Figure 6:**
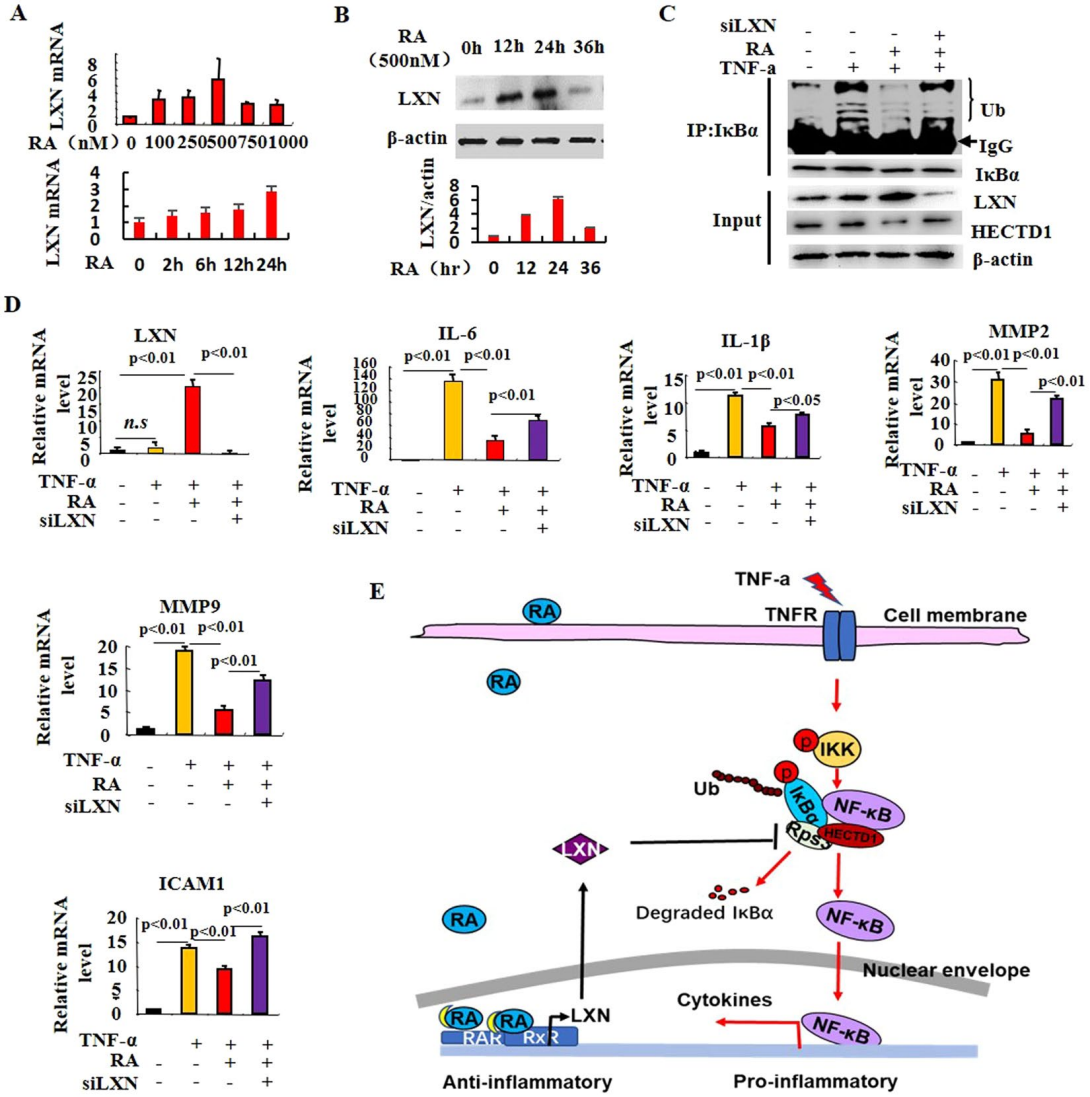
Retinoic acid represses TNF-α-induced inflammatory response by induction of LXN. (**A**) Time-dependent and dose-dependent effect of retinoic acid on LXN expression in HIEC cells as determined by qPCR. (**B**) Time-dependent effects of retinoic acid (500 nM) on LXN expression was determined by Western blot analysis. (**C**) 48 h after transfection of LXN siRNA, cells were treated with retinoic acid (500 nM) for 24 h. Before harvested, the cells were stimulated with TNF-α (20 ng/mL) for 30 min, and protein samples were subjected to immunoprecipitation with anti-IκBα antibody followed by immunoblot analysis with anti-ubiquitin antibody to detect ubiquitylation of IκBα. (**D**) Effect of retinoic acid and LXN on the expression of cytokines in HIEC cells, determined by qPCR. (**E**) Proposed model for LXN mediating the anti-inflammatory process. TNF-α induces inflammatory response, as well as, promotes the interaction of Rps3 and HECTD1, which enhances the ubiquitylation of IκBα and further activation of NF-κB; retinoic acid increases LXN expression, and the expression of LXN competitively binds with Rps3 and dissociates the interaction between Rps3 and HECTD1, which leads to decreasing the ubiquitylation of IκBα, and eventually inhibits the inflammatory response.

Retinoic acid (RA) has been widely used to treat various inflammatory and cancer-related diseases, including colitis and colon cancer. Importantly, RA has been reported to induce the expression of LXN in prostate epithelial cell lines^21^. To examine whether pharmacological activation of LXN could affect inflammatory response, the expression of LXN was determined when cells were treated with RA. As shown in Fig. 6A, at the concentration of 100 nM, RA induced LXN mRNA expression by approximately 3-fold. The maximal induction of RA on LXN mRNA expression was observed at the concentration of 500 nM, with an increased LXN expression by approximately 6-fold (Fig. 6A). The time course of LXN expression, when incubated with 500 nM RA, showed a maximal induction of LXN expression at 24 hr upon RA stimulation, as well as, the expression of LXN at protein level also was examined by western blot (Fig. 6B). These results indicate that LXN play potential role in the pharmacological process of RA. We next assessed whether the TNF-α-induced IκBα ubiquitylation is impacted by RA stimulation. As shown in Fig. 6C, TNF-α increases the ubiquitylation of IκBα, and such ubiquitylation was dramatically impaired when the cells were treated with RA, as well as the increasing of LXN expression. However, the efficiency inhibitory of RA on IκBα ubiquitylation was reversed when LXN was knockdown, further indicating the critical role of LXN in NF-ĸB mediated inflammatory response. We also analyzed the effect of RA on the production of inflammatory cytokines both in normal and LXN knockdown cells under TNF-α stimuli, by qPCR. We confirmed that LXN was significantly induced by RA (Fig. 6D). The expression of interleukins (such as IL-1β, IL-6), matrix metalloproteinases (such as MMP2 and MMP9) and cell adhesion molecules (ICAM1) induced by TNF-α were significantly inhibited by RA treatment, however, were dramatically reversed when LXN was knockdown (Fig. 6D). Taken together, these results strongly support the conclusion that RA attenuates TNF-α induced inflammatory response by induction of endogenous LXN (Fig. 6E).

### LXN is required for the therapeutic effect of retinoic acid on colitis in DSS-induced mice

**Figure 7:**
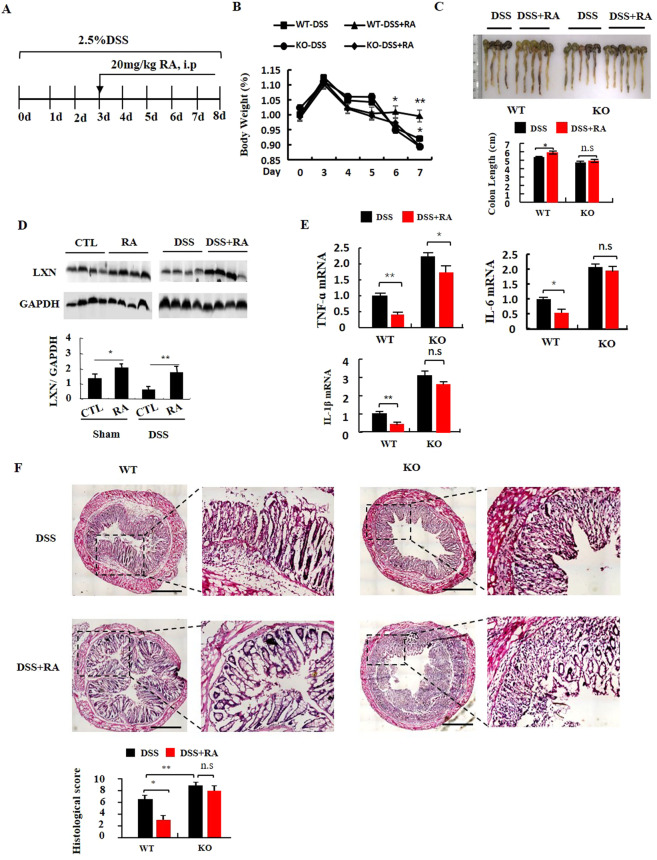
LXN is required for the therapeutic effect of retinoic acid on DSS colitis. (**A**) Experimental protocol for the induction of the acute colitis model and treated with 20 mg/kg retinoic acid in LXN^−/−^ (KO) and littermate LXN^+/+^ (WT) mice. **(B)** Changes in body weight of DSS induced WT and KO mice without or with retinoic acid treated (relative to starting weight, set as 100%) is shown (n = 12). (**C**) The gross morphology of shortened colon was photograph and the colon length was measured at day 7 (n = 4-6). (**D**) The expression of LXN in colon tissue in WT mice treated with retinoic acid was determined by Western blot. (**E**) The expression of cytokines in colon tissues from DSS induced WT and KO mice treated with retinoic acid were determined by qPCR (n = 6). (**F**) Representative images of H&E-stained colons from DSS-induced WT and KO mice treated with retinoic acid are shown. Scale bars = 100 μm. The results shown are the mean ± SEM. A two-tailed unpaired t-test was used to compare experimental groups. *p < 0.05; **p < 0.01; n.s, no significant.

Bai and colleagues showed that treatments of mice with RA ameliorated disease in the TNBS colitis model^22^. Hong and colleagues showed similar efficacy for RA in the mouse model of DSS-induced colitis^23^. We found that LXN could be induced by RA in HIECs, and thus repressing TNF-α-induced inflammatory response (Fig. 6). We hypothesize that LXN may play a role in the treatment of colitis with retinoic acid. To this end, we induced colitis by administering 2.5% DSS in drinking water and treated the mice with RA (Fig. 7A). DSS administration was associated with significant body weight loss in both WT and LXN^−/−^ mice. However, treatment with RA resulted in significant amelioration of colitis in WT mice, but not in LXN^−/−^ mice, as shown by an increase body weight (Fig. 7B), colon length (Fig. 7C), improvement in splenomegaly (Fig. S5) and reduced rectal bleeding (data not shown). Western blot shown that RA treated significantly increase the expression of LXN in mice colon tissue (Fig. 7D). The expression of cytokines in colon were determined by qPCR. As shown in Fig. 7E, RA treatment significantly decreased the levels of TNF-α, IL-6, and IL-1β in colon tissue in WT mice during DSS-induced colitis, but the effect is not obvious in LXN^−/−^ mice. Finally, we evaluated the colitis pathological sections form WT and LXN^−/−^ mice treated with retinoic acid. Histological analysis of the colons shown that mice (both WT and LXN^−/−^) with DSS-induced colitis exhibit mucosal conges-tion, erosion, loss of goblet cells. However, the colonic tissue damage of the WT mice treated with RA was significantly improved, while the therapeutic effect of RA in LXN^−/−^ mice was very insignificant (Fig. 7F). The data indicate that LXN is required for the therapeutic effect of retinoic acid on DSS colitis in mice.

## Discussion

LXN is implicated in the inflammatory response^13,24,25^. However, the functional roles of LXN in inflammatory are less well understood, and no data are available regarding the role of LXN in colitis. In this study, our findings highlight an inverse correlation between LXN and colitis. We reveal that LXN deficiency leads to the severity of mice colitis induced by DSS, and ectopic LXN attenuates inflammatory response by inhibiting the ubiquitination and degradation of IκBα. Our findings highlight a correlation between LXN and colitis via HECTD1/Rps3/IκBα pathway. This report is the first to demonstrate the functional role of LXN in colitis.

IBD is an inflammatory condition of the gastrointestinal tract, in which several factors, genetic, environmental, as well as the fine interplay between intestinal microbiota and related host immune response, contribute to its pathogenesis, in which NF-κB signaling pathway plays a key role in the development of IBD^26–29^. Many signals that lead to cell growth, differentiation and apoptosis could activate IκBα transcription factors, and further regulate the expression of inflammatory-related cytokines. A variety of animal models have been widely used to study the etiology, pathogenesis and test the efficacy of newly developed drugs of IBD, especially DSS-induced colitis model is the most widely used^23,30–32^. In animal model, we found that LXN deficiency leads to severity of DSS colitis, which can be demonstrated by a number of clinicopathological indicators, including more severe weight loss, hematochezia, shortening of the colon and rectum, damage to the mucosa and loss of goblet cells, increased proinflammatory cytokines and increased STAT3 activity. Many literatures have reported the key role of JAK/STAT3 signal transduction pathway in the pathogenesis of IBD^33–36^. In the cell model, we prove that ectopic expression of LXN inhibits both basal and TNF-α-induced NF-κB-Luc activity. Many inflammatory cytokines could be inhibited by ectopic expression of LXN, however, enhanced by LXN knockdown. Interestingly, we found that ectopic expression of LXN attenuates both the basal and TNF-α induced the degradation of IκBα and results to the accumulation of IκBα in HIECs. These results suggest that LXN may regulate the inflammatory response of intestinal epithelial cells through the NF-κB pathway.

A growing body of evidence suggests that Rps3 involves in the regulation of NF-κB activity^15–17^. Rps3 is a 26 kDa protein that shuttles between the cytoplasm and the nucleus. Stanborough *et al*. reported that Rps3 interacts with IκBα in resting cells, and maintenance of an Rps3 pool for the NF-κB pathway^17^. Recently, Liang and colleagues reported that LXN associates with Rps3 and regulates cell-cycle arrest and cell death in leukemogenic cells^14^. We confirmed the interaction between Rps3 and IκBα in colon cells, and prove that this interaction is functional, because this interaction could be induced by TNF-α stimuli and be disrupted by ectopic expression of LXN, however, enhanced by LXN knockdown (Fig. 3F,G). In addition, knockdown of Rps3 leads to decreasing ubiquitylation of IκBα (Fig. 5F), indicating that Rps3 is required for the ubiquitylation of IκBα.

We further address the functional roles of LXN in colon cells by proteomics strategy. We identified HECTD1 as LXN associating protein. Because HECTD1 is an E3 ubiquitin ligase, which widely involved in the regulation of cancer invasion^37,38^, this prompted us to hypothesis that HECTD1 may be related to the ubiquitylation of IκBα. Interestingly, HECTD1 may interact with adenomatous polyposis coli (APC), whose dysfunction has been linked to the development of most forms of colorectal cancer^39^. We confirmed that LXN, HECTD1 and Rps3 form a functional complex (Fig. 4). LXN and HECTD1 competitively interacted with Rps3: on the one hand, Rsp3 knockdown attenuates the interaction between LXN and HECTD1 (Fig. 5A); on the other hand, LXN deficiency enhances the interaction of HECTD1 and Rps3 (Fig. 5B). As expectedly, we prove that IκBα is one of substrate of HECTD1 (Fig. 5C–E), and Rps3 is required for HECTD1 to targeting and Lys48-linked polyubiquitination of IκBα (Fig. 5F–H). Thereby, our data have provided new insight into the mechanism of LXN modulating inflammatory responses in colon cells.

All-trans retinoic acid (RA) is well known as an anti-IBD and anti-tumor agent in clinical^22,23,40–43^. Hong *et al*. reported that RA attenuates experimental colitis through inhibiting NF-κB activation, however, its mechanism is very unclear^23^. Putative binding sites for RA responsive elements (RAREs) are present within the upstream of LXN transcription start sites^21^. Oldridge *et al*. reported that LXN could be induced by RA in prostate epithelial cell lines^21^. Therefore, we speculate whether LXN mediates the anti-inflammatory effect of RA in colitis. Indeed, we found that RA could indeed induce LXN expression in HIECs (Fig. 6A,B). Furthermore, RA treatment significantly inhibits TNF-α induced IκBα ubiquitylation and cytokines production, however, this inhibitory effect was reversed in LXN knockdown HIECs (Fig. 6C,D). Finally, we treated DSS colitis mice with RA. Compared with LXN^−/−^ mice, RA has better therapeutic effect on WT DSS colitis mice, further demonstrating that LXN mediates the anti-inflammatory process of RA.

There are also shortcomings in our research. Firstly, *in vivo* study was performed only on DSS-induced colitis model. It would be more meaningful to test this finding in another model of colitis. In this study, we believe that this model has been able to demonstrate the important role of LXN in the development of colitis. Secondly, many studies have shown that LXN is involved in the proliferation and apoptosis of hematopoietic stem cells. Liang *et al*. reported the function of LXN in hematopoietic stem cells (HSCs), they revealed that LXN expression in the hematopoietic compartment was demonstrated to negatively regulate the number of HSCs, which indicated LXN is a potential cancer suppressor^44^. In this study, we did not detect the effect of LXN on the infiltration of immune cells during the development of DSS colitis, nor did we further observe whether the LXN deficiency affects the occurrence of colorectal cancer. Of course, these studies are being carried out in our laboratory.

In summary, this study shows that LXN is a critical regulator in colitis. The mechanisms underlying LXN form a complex with HECTD1, IκBα and Rps3, and this complex catalyzes the Lys48-linked polyubiquitination of IκBα. LXN deficiency enhances the interaction of HECTD1 and Rps3, contributing to the ubiquitination degradation of IκBα. Thus, we provide a novel mechanism by which LXN modulates colitis and significant implications for the development of novel strategies for the treatment of IBD by targeting LXN.

## Supplementary information

Supplementary Data-SR-R1.
