# Author Correction: Latexin deficiency in mice up-regulates inflammation and aggravates colitis through HECTD1/Rps3/NF-κB pathway

**DOI:** 10.1038/s41598-025-18604-8

**Published:** 2025-09-17

**Authors:** Yaping Li, Baohua Huang, Hua Yang, Shuang Kan, Yanling Yao, Xin Liu, Shiming Pu, Guozhang He, Taj-Malook Khan, Guangying Qi, Zuping Zhou, Wei Shu, Ming Chen

**Affiliations:** 1 State Key Laboratory for Chemistry and Molecular Engineering of Medicinal Resources, School of Chemistry and Pharmacy, Guangxi Normal University, Guilin, 541004 People’s Republic of China; 2 Central Laboratory, The Affiliated Yantai Yuhuangding Hospital of Qingdao University, Yantai, 264000 People’s Republic of China; 3 School of Life Sciences, Research Center for Biomedical Sciences, Guangxi Normal University, Guilin, 541004 People’s Republic of China; 4 School of Basic Medical Science, Guilin Medical University, Guilin, 541004 People’s Republic of China; 5 College of Biotechnology, Guilin Medical University, Guilin, 541004 People’s Republic of China

Correction to: *Scientific Reports* 10.1038/s41598-020-66789-x, published online 17 June 2020.

This Article contains errors.

Due to an error in the data organization during figure assembly the actin western blot from Figure 3E was reused in Figure 6B. As the Authors were unable to locate the corresponding actin western blot for Figure 6B, they newly repeated the experiment and provided actin and LXN replacement blots for Figure 6B. Consequently, the quantification in Figure 6B was also redone to represent the new blots.

The correct Figure 6 and accompanying legend appear below.

**Fig. 6:**
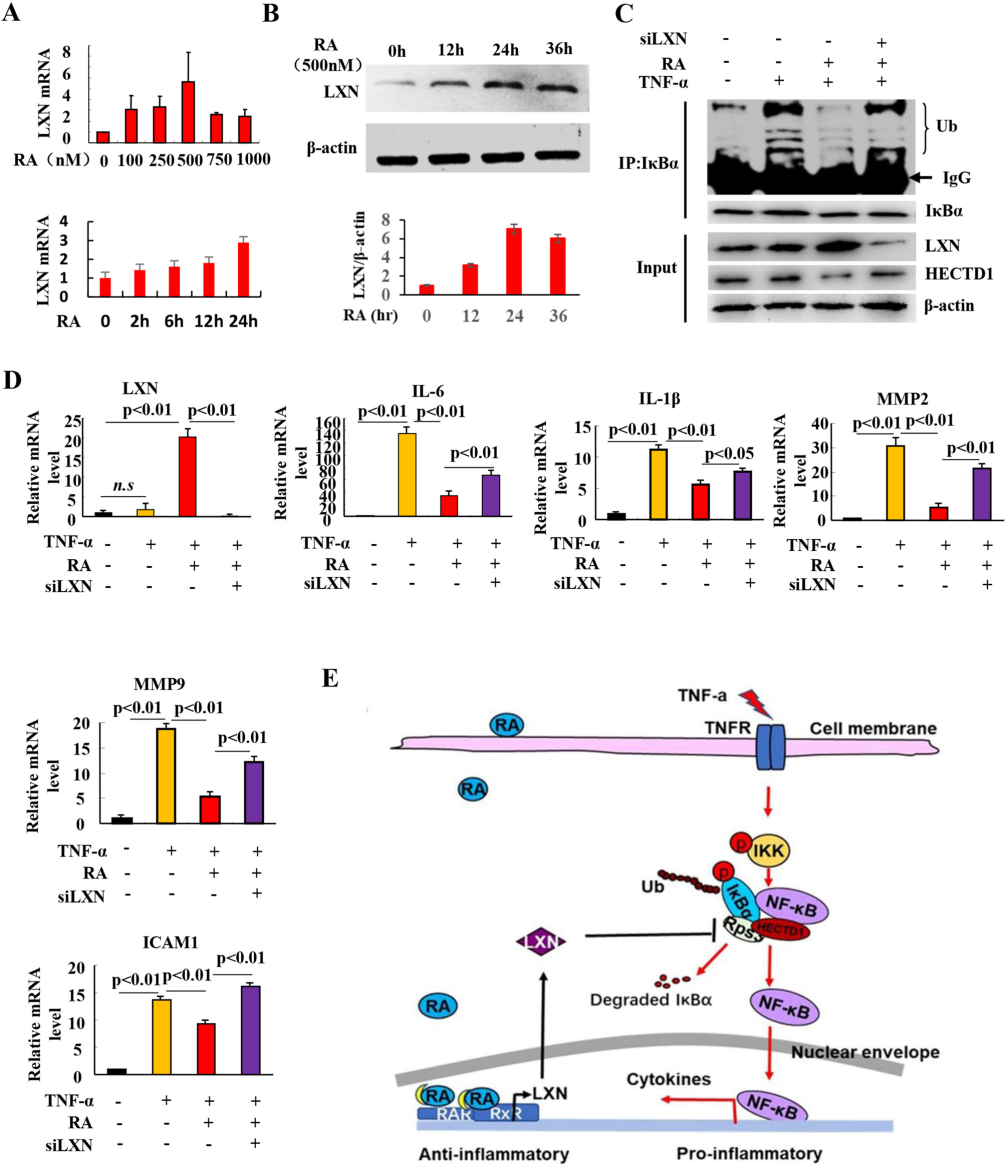
Retinoic acid represses TNF-α-induced inflammatory response by induction of LXN. **A** Time-dependent and dose-dependent effect of retinoic acid on LXN expression in HIEC cells as determined by qPCR. **B** Time-dependent effects of retinoic acid (500 nM) on LXN expression was determined by Western blot analysis. **C** 48 h after transfection of LXN siRNA, cells were treated with retinoic acid (500 nM) for 24 h. Before harvested, the cells were stimulated with TNF-α (20 ng/mL) for 30 min, and protein samples were subjected to immunoprecipitation with anti-IκBα antibody followed by immunoblot analysis with anti-ubiquitin antibody to detect ubiquitylation of IκBα. **D** Effect of retinoic acid and LXN on the expression of cytokines in HIEC cells, determined by qPCR. **E** Proposed model for LXN mediating the anti-inflammatory process. TNF-α induces inflammatory response, as well as, promotes the interaction of Rps3 and HECTD1, which enhances the ubiquitylation of IκBα and further activation of NF-κB; retinoic acid increases LXN expression, and the expression of LXN competitively binds with Rps3 and dissociates the interaction between Rps3 and HECTD1, which leads to decreasing the ubiquitylation of IκBα, and eventually inhibits the inflammatory response.

