# Supplementary material for: Latexin deficiency in mice up-regulates inflammation and aggravates colitis through HECTD1/Rps3/NF-κB pathway
Source: Sci Rep. 2020 Jun 17;10:9868. doi: 10.1038/s41598-020-66789-x (PMC7299958; doi:10.1038/s41598-020-66789-x)
Supplement: Supplementary file 1 — Supplementary Data-SR-R1. [file 41598_2020_66789_MOESM1_ESM.pdf]

Latexin deficiency in mice up-regulates inflammation and aggravates colitis through HECTD1/Rps3/NF-κB pathway

Yaping Li[1#], Baohua Huang[1#], Hua Yang[1#], Shuang Kan[1], Yanling Yao[1], Xin Liu[2], Shiming Pu[3], Guozhang He[1], Taj-Malook Khan[1], Guangying Qi[4], Zuping Zhou[3], Wei Shu[5*], Ming Chen[1*]

[1]State Key Laboratory for Chemistry and Molecular Engineering of Medicinal Resources, School of Chemistry and Pharmacy, Guangxi Normal University, Guilin, 541004, P. R. China;

[2]Central Laboratory, The Affiliated Yantai Yuhuangding Hospital of Qingdao University, Yantai, 264000, P.R. China;

[3]School of Life Sciences, Research Center for Biomedical Sciences, Guangxi Normal University, Guilin, 541004, P. R. China；

[4]School of Basic Medical Science, Guilin Medical University, Guilin, 541004, P.R. China；

[5] College of Biotechnology, Guilin Medical University, Guilin, 541004, P.R. China；

Supplementary Data

Methods

Pathological evaluation. Disease severity or disease activity index (DIA) of colitis-associated disease mouse model was determined based on weight loss, blood in the stool, and stool consistency. The intensity of colitis was monitored and the clinical parameters were evaluated as fellow: anal erosion (score 0-3: 0, normal; 1, mild; 2, moderate; 3, severe), anal bleeding (score 0-3: 0, normal; 1, mild; 2, moderate; 3, severe), diarrhea (score 0-3:0, normal; 1, mild; 2, moderate; 3, severe)**.** Histology was scored in a blinded manner by pathologists. The degree of inflammation was scored as follows: on a scale of 0-3 (0, negative; 1, mild; 2, moderate; 3, severe); damage in crypt architecture was scored as follows: on a scale of 0-4 (0, negative; 1, 0-30% damage to epithelium; 2, 31-65% damage to epithelium; 3, structurally defective epithelium; 4, loss of crypt and epithelium destruction). Tumor grade was assessed by a pathologist blinded to the mouse genotype and treatment, using clinical and pathological scores as described previously (Wirtz S, et al. Nature protocols 2007; Kargl J, et al. Journal of molecular medicine 2013). The total histopathological score was determined by the summation of the scores from each category.

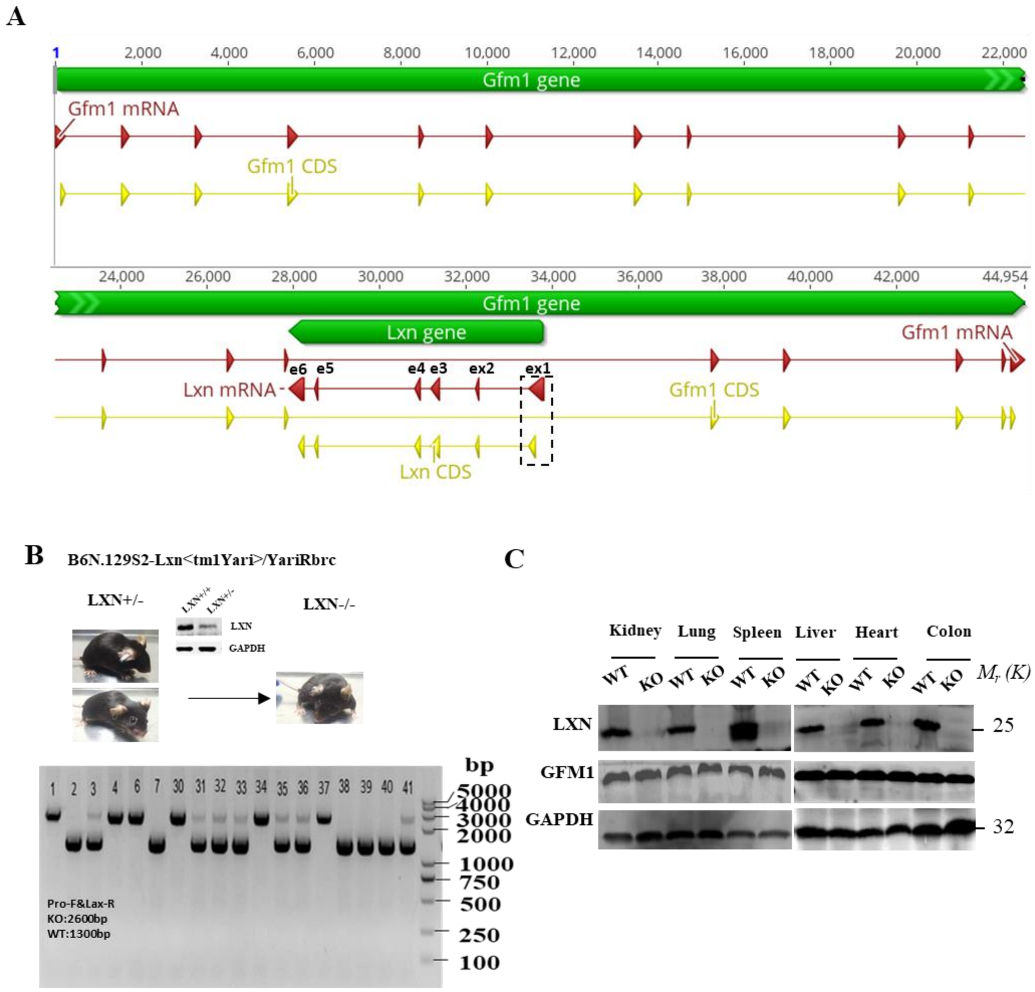

Fig S1. Generation of LXN-deficient mice. (A) Location of LXN and GFM1 genes in the genome. The coding region of LXN is located in the intron region of GFM1 gene. The coding region of exon 1 of the LXN gene was replaced by a neomycin resistant cassette in the target vector. Green thick lines: LXN and GFM1 genes; Red thin lines: LXN and GFM transcripts; Colored triangles: exons; Yellow thin lines: CDS. **(B)** The heterozygous pairs were used to generate homozygous LXN-/- (KO) and littermate wild-type (WT) mice. Mouse genotype was determined by PCR using the primers as below: Pro-F, 5'-CGTTAGACTTTAAAATGCTCACTTTGGAAGCCCATACT-3'; Lax-R, 5'-CCTCCTTGCTGGCCTGCTGGACCGTCTGCACC-3'. These probes recognize 2600bp fragment in the homozygous mice (LXN-/-), 1300bp fragment in wild-type mice (LXN+/+), and both fragments in heterozygous mice (LXN+/-). **(C)** Western blot of LXN and GFM1 expression in the wild-type (WT) and homozygous (KO) mice.

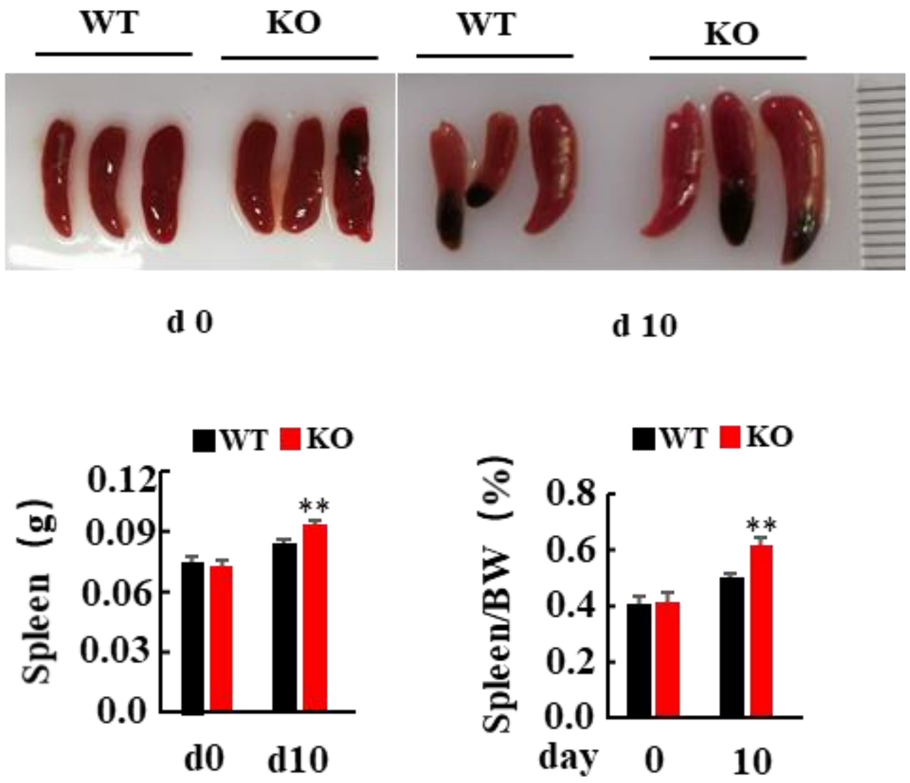

Fig S2. LXN deficiency aggravates DSS-induced splenomegaly in mice.

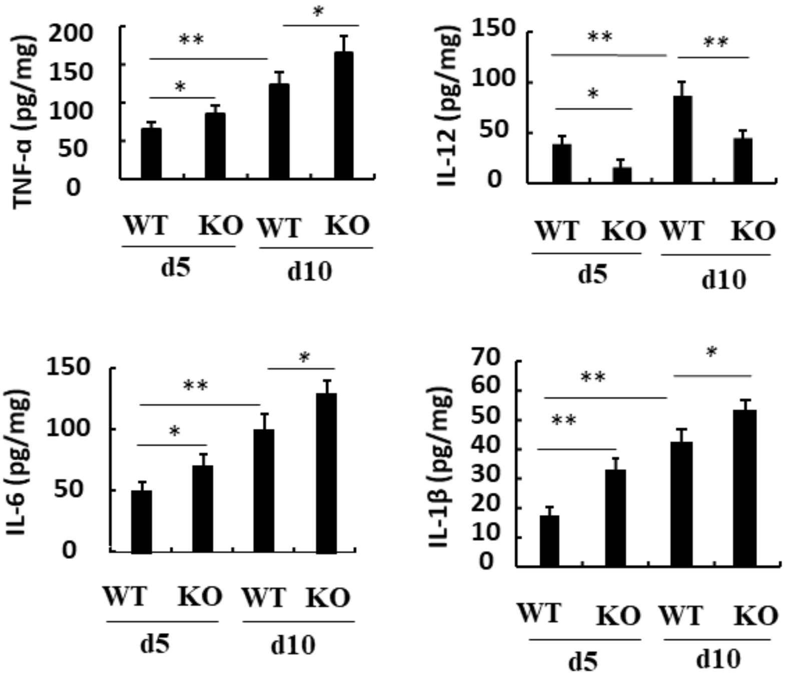

Fig S3. LXN deficiency increases the expression of proinflammatory factors in colorectal tissue in DSS-induced mice.

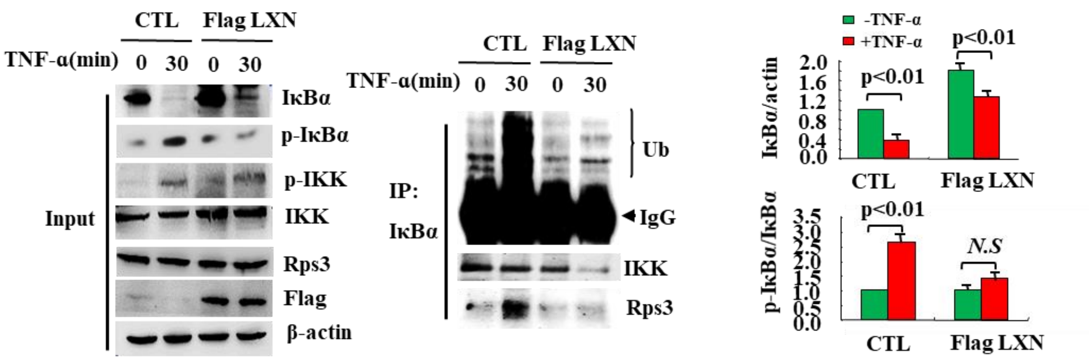

Fig S4. Overexpression of LXN inhibits the TNF-α induced phosphorylation of IκBα, but has no effect on the phosphorylation of IKK. HCT116 cells were transfected with Flag-LXN plasmid. 48 h after transfection, cells were treated with TNF-α (20 ng/mL) for 30min, Western blot was performed with antibody as indicated. For immune-precipitation, anti-IκBα antibody was used, and the immune-precipitate complex was separated by 10% SDS-PAGE, followed by immunoblotting with antibody as indicated.

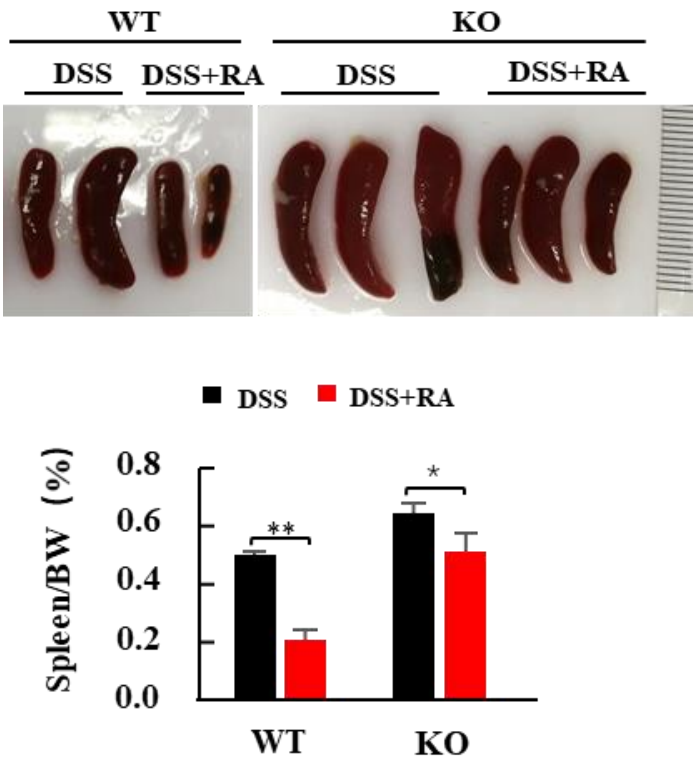

Fig S5. Retinoic acid treatment significantly alleviates DSS induced splenomegaly in WT mice, but has limited effect on LXN KO mice.

Fig S6. Unprocessed scans of immunoblots shown in the main figures.

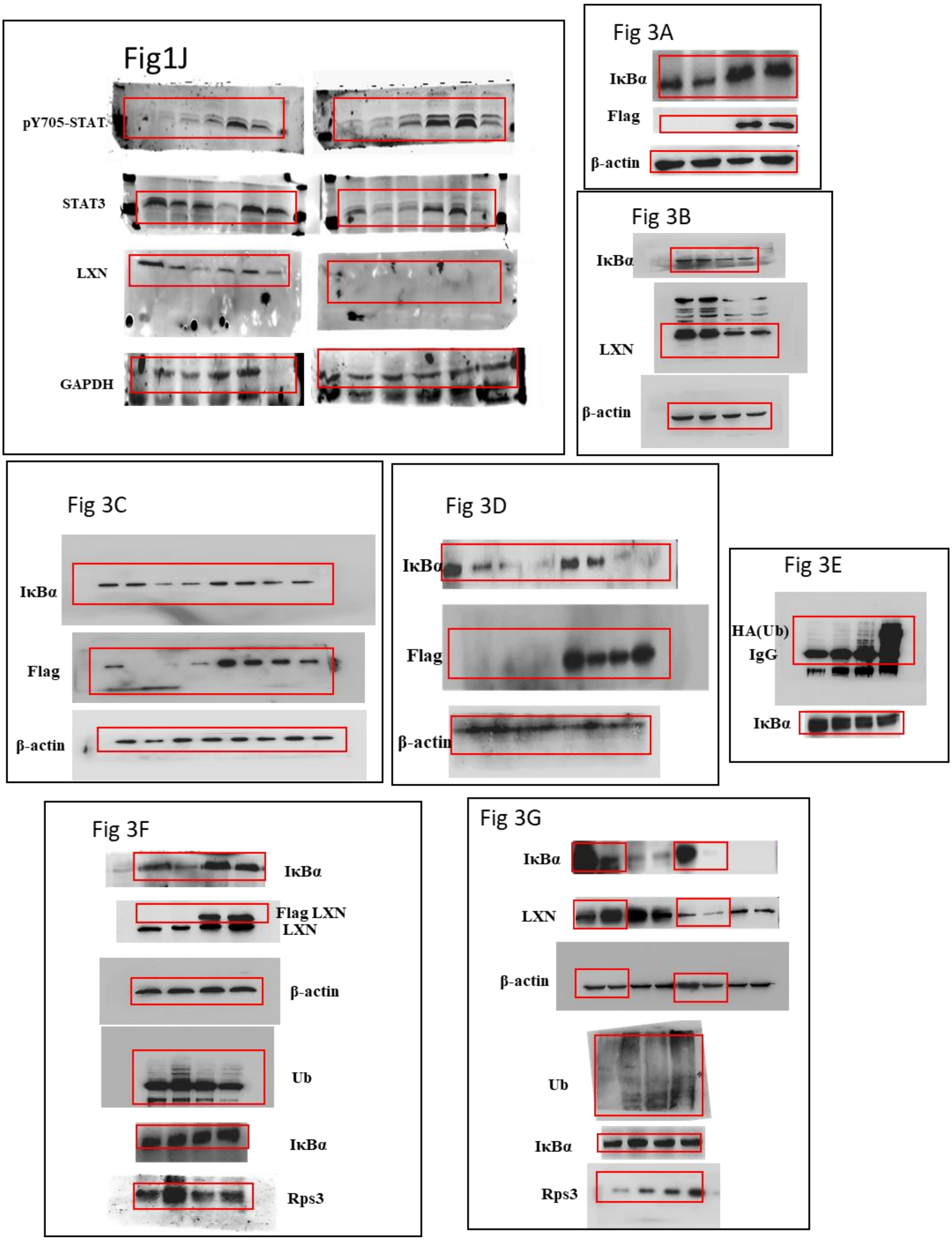

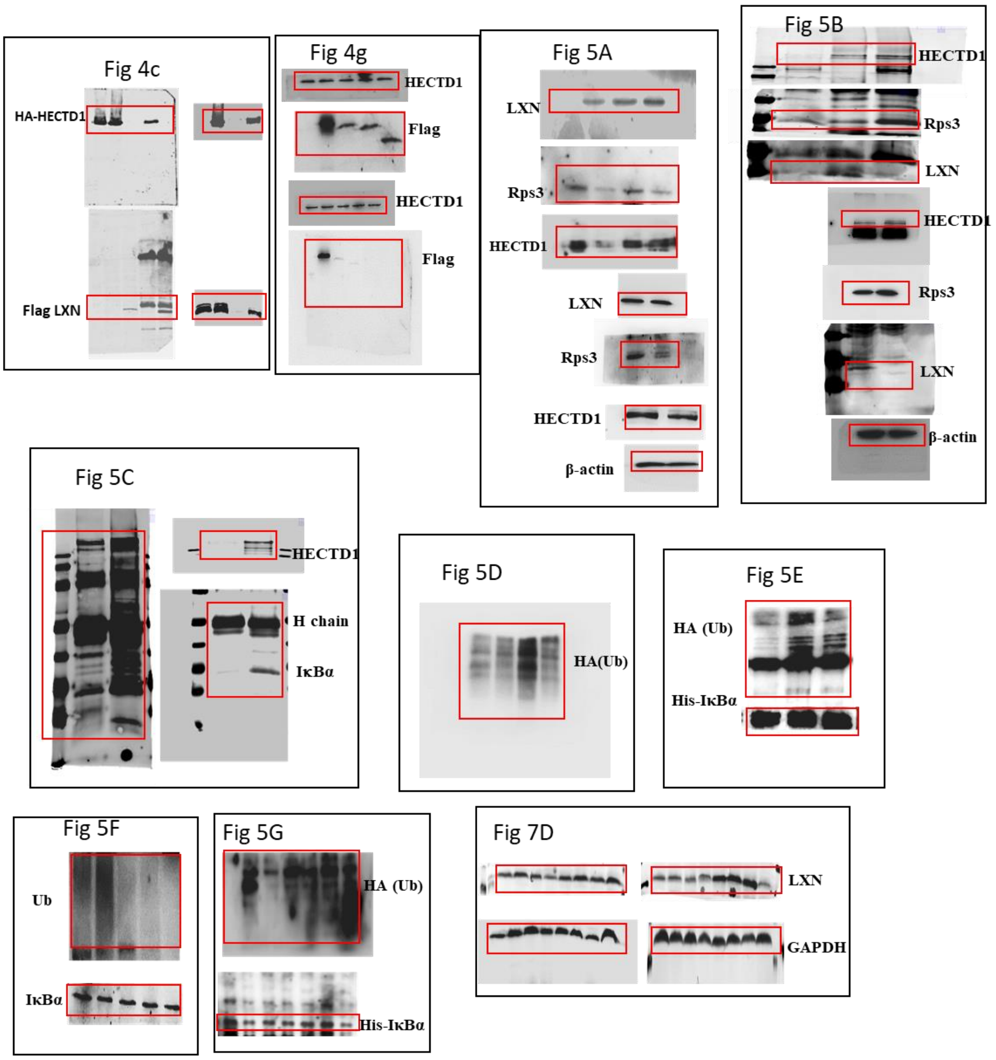

Table S1. Information of antibodies

Name	Supplier	Catalog number/or clone	WB dilution	IF dilution	IHC dilution	IP
LXN	Sino Biological	10211-R101	1:1000	1:200	1:100	1:100
IκBα	Sino Biological	12045-MM03	1:1000			1:250
GAPDH	ZSGB-BIO	TA-08	1:1000			
β-actin	ZSGB-BIO	TA-09	1:1000			
p65	Beyotime	AF2046	1:1000	1:200		

**IgG**	**Beyotime**	**A7016**				**1:100**
**Flag**	**Sino Biological**	**101274-MM05**	**1:1000**			**1:40**
**Myc**	**Sino Biological**	**100029-MM08**	**1:5000**			
**HA**	**CST**	**#3724**	**1:1000**			**1:50**
**His**	**CST**	**#2365**	**1:1000**			**1:25**
**Ubiquitin**	**Beyotime**	**AF0306**	**1:1000**			**1:100**
**Rps3**	**Sino Biological**	**101346-T38**	**1:1000**	**1:200**		
**HECTD1**	**abcam**	**ab101992**	**1:2000**	**1:500**	**1:200**	**2μg/mg of lysate.**
**K48-linkage Specific Polyubiquitin**	**CST**	**#42895**	**1:1000**			
**STAT3**	**eBioscience**	**9D8**	**1:5000**			
**pY705-STAT3**	**Beyotime**	**PA5-85445**	**1:2000**	**1:200-**		
**pS727-STAT3**	**Beyotime**	**44-384G**	**1:2000**			

Table S2. Primer for qPCR

**Primer**	**Sequence (5’-3’)**
LXN (h)	Forward, 5′-ACAGAACTACATCAACTACCAGC-3′<br>Reverse, 5′-GTGATACTTATGTCCTCTTCCTGG-3′
IL-1β (h)	Forward, 5′-ATGGACAAGCTGAGGAAGATG-3′<br>Reverse, 5′-CCCATGTGTCGAAGAAGATAGG-3′
IL-12 (h)	Forward, 5′-TACACCAGCAGCTTCTTCATC-3′<br>Reverse, 5′-CCACCTGCCGAGAATTCTTTA-3′
IL-6 (h)	Forward, 5′-CCACTCACCTCTTCAGAACG-3′<br>Reverse, 5′-CATCTTTGGAAGGTTCAGGTTG-3′
IL-8 (h)	Forward, 5′-CTTGGCAGCCTTCCTGATTT-3′

	Reverse 5′-GGGTGGAAAGGTTTGGAGTATG-3′
ICAM1 (h)	Forward, 5′-CAATGTGCTATTCAAACTGCCC-3′<br>Reverse, 5′-CAGCGTAGGGTAAGGTTCTTG-3′
VCAM1 (h)	Forward, 5′-TCTACGCTGACAATGAATCCTG-3′<br>Reverse, 5′-AGGGCCACTCAAATGAATCTC-3′
18S (h)	Forward, 5′-TCAAGAACGAAAGTCGGAGG-3′<br>Reverse, 5′-GGACATCTAAGGGCATCAC-3′
TNF-α(m)	Forward,5’-ACCCTCACACTCAGATCATC-3’<br>Reverse,5’-GAGTAGACAAGGTACAACCC-3’
IL-6(m)	Forward,5’-TACCACTTCACAAGTCGGAGGC-3’<br>Reverse,5’-CTGCAAGTGCATCATCGTTGTTC-3’
IL-1β (m)	Forward,5’-TGCCACCTTTTGACAGTGATG-3’<br>Reverse,5’-TGATGTGCTGCTGCGAGATT-3’
GAPDH(m)	Forward,5’-AGGTCGGTGTGAACGGATTTG-3’<br>Reverse,5’-TGTAGACCATGTAGTTGAGGTCA-3’

Table S3. Information of siRNA

**Gene symbol**	**Protein**	**Sequence#1**	**Sequence#2**
SASI_Hs01_00073945	LXN	5′-GGAAGUACAACUGGAAUAAdTdT-3′	5′-UUAUUCCAGUUGUACUUCCdTdT-3′
SASI_WI_00000070	HECTD1	5′-GGGUGGAGACAUGUGAGAAdTdT-3′	5′-UUCUCACAUGUCUCCACCCdTdT-3′
SASI_Hs01_00243869	Rps3	5′-GACAGAGGGCUAAAUCCAUdTdT-3′	5′-AUGGAUUUAGCCCUCUGUCdTdT-3′
